# A New Approach for Testing Fetal Heart Rate Monitors

**DOI:** 10.3390/s20154139

**Published:** 2020-07-25

**Authors:** Daniele Bibbo, Tomas Klinkovsky, Marek Penhaker, Petr Kudrna, Lukas Peter, Martin Augustynek, Vladimír Kašík, Jan Kubicek, Ali Selamat, Martin Cerny, Daniel Bielcik

**Affiliations:** 1 Department of Engineering, University of Roma Tre, Via Vito Volterra, 62, 00146 Rome, Italy; 2 Department of Cybernetics and Biomedical Engineering, Faculty of Electrical Engineering and Computer Science, VSB—Technical University of Ostrava, 17. listopadu 2172/15, 708 00 Ostrava-Poruba, Czech Republic; tomas.klinkovsky@vsb.cz (T.K.); marek.penhaker@vsb.cz (M.P.); lukas.peter@vsb.cz (L.P.); martin.augustynek@vsb.cz (M.A.); vladimir.kasik@vsb.cz (V.K.); jan.kubicek@vsb.cz (J.K.); martin.cerny@vsb.cz (M.C.); daniel.bielcik@vsb.cz (D.B.); 3 Department of Biomedical Technology, Faculty of Biomedical Engineering, Czech Technical University in Prague, nam. Sitna 3105, 272 01 Kladno, Czech Republic; petr.kudrna@fbmi.cvut.cz; 4 Malaysia-Japan International Institute of Technology (MJIIT), Universiti Teknologi Malaysia, Jalan Sultan Yahya Petra, 54100 Kuala Lumpur, Malaysia; aselamat@utm.my

**Keywords:** fetal heart rate, cardiotocograph, doppler effect, heart movement simulator, tests of medical device, fetal heart rate monitor device

## Abstract

In this paper, a new approach for the periodical testing and the functionality evaluation of a fetal heart rate monitor device based on ultrasound principle is proposed. The design and realization of the device are presented, together with the description of its features and functioning tests. In the designed device, a relay element, driven by an electric signal that allows switching at two specific frequencies, is used to simulate the fetus and the mother’s heartbeat. The simulator was designed to be compliant with the standard requirements for accurate assessment and measurement of medical devices. The accuracy of the simulated signals was evaluated, and it resulted to be stable and reliable. The generated frequencies show an error of about 0.5% with respect to the nominal one while the accuracy of the test equipment was within ±3% of the test signal set frequency. This value complies with the technical standard for the accuracy of fetal heart rate monitor devices. Moreover, the performed tests and measurements show the correct functionality of the developed simulator. The proposed equipment and testing respect the technical requirements for medical devices. The features of the proposed device make it simple and quick in testing a fetal heart rate monitor, thus providing an efficient way to evaluate and test the correlation capabilities of commercial apparatuses.

## 1. Introduction

During pregnancy, the development of the different organs usually follows phases that start in different weeks. Among these, the heart begins to work at the fourth week of life, so, from this stage, the fetus starts to use its own bloodstream [1]. The fetal heart activity can be monitored from the seventh week using specific devices, such as ultrasound-based ones [2]. Starting from the 20th week, the fetal heartbeats can be heard without amplification, and the corresponding Fetal Heart Rate (FHR) normally ranges from about 110 to 150 beats per minute (BPM) [3]. It is important to monitor the heart activity as soon as possible and with accurate and reliable systems, especially by using ultrasound technics [4,5], in order to timely detect any abnormal behavior (e.g., cardiorespiratory disease).

For fetus heart rate measurement, FHR monitor devices or combined cardiotocography (CTG) monitors are usually adopted. These combine measurements by one tocographic (TOCO) and at least one ultrasound (US) probe usually recorded in the last pregnancy trimester period. The FHR monitor device can be a valid and reliable system to monitor cardiac activity at its very beginning, with a high degree of sensitivity [6,7,8,9,10].

The CTG allows both early diagnoses of uterine disorders and hypoxemic changes that can compromise the fetus growth (e.g., twins or triplets). In general, FHR monitor cannot directly detect the presence of fetal hypoxia, except for extreme situations, but it is used to detect frequency changes induced by barochemoreceptors in large myocardial vessels [11]. This system responds to changes in fetal vessels to a deeper degree of respiration [12,13,14].

An FHR registration is based on the acquisition of the heart rate cardiographic curve considering two consecutive cycles [15]. Under normal conditions, the interval occurring between consecutive cycles changes very slightly. 

FHR monitoring is performed for a duration that is in the range from 1 to 10 min (3 min at bradycardia) and using these data the tachogram, which shows the heart rate variability over time, is normally computed for diagnosis [16]. If fetal records are interrupted, either contractions or stress tests define the basal heart rate as the mean heart rate per minute. Where appropriate, periodic accelerations or decelerations can occur [17,18,19].

Furthermore, it is possible to acquire fetus heart rate by acoustic sensing [20,21], and the most common FHR monitor devices rely on the Doppler effect [8]. 

**Figure 1:**
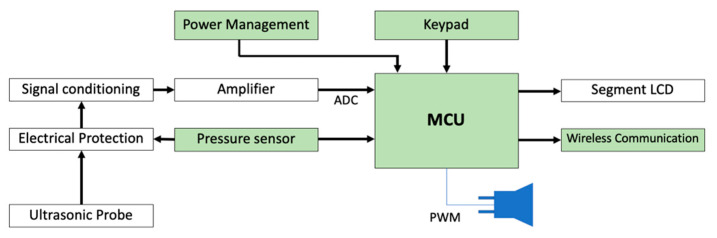
The general scheme of Doppler measuring instruments for heart rate.

A Doppler cardiotocograph, whose general scheme is reported in Figure 1, is based on the use of ultrasonic waves [22], generated by the oscillator embedded in the probe. Those are sent towards the heart, and, due to its movement when a beat occurs, an associated Doppler effect is generated that changes their frequency. The signal, after being properly conditioned in a first analog block of the measurement chain, is acquired and processed by the microprocessor unit (MCU) that using the correlation function extracts the information related to cardiac activity, providing also to display it on the LCD screen, to the associate sound generation and to the communication to a remote listener using an encrypted wireless system. In a medical device, the electrical protection stage, used to guarantee the safety conditions to the patient if a fault occurs by isolating the probe from the electrical network, is always mandatory.

While many approaches have been developed, and proposed also in the commercial field, to simulate the electrical signal associated with the heart activity [23,24], to the best of our knowledge, no hardware solutions are available to simulate the mechanical/acoustic effect of the fetal heart activity.

Currently, both in research and in the commercial field many simulators are available for testing a wide range of medical devices. Concerning the heart activity, most available simulators are based on the reproduction of electrophysiological signals related to the heart electrical activity, including the simulators for the ECG of the fetus. These systems cannot help in testing CTG probes, since, for example, a transducer from electrical to mechanical/acoustic signals is strictly needed to do this.

In previous works, many possibilities of simulating the fetal heart rate have been exploited using mainly a software approach, based on the modeling of this signal, also to calibrate and test the accuracy of these systems [25].

All proposed approaches aim to obtain a valid and reliable technique to test a fetal cardiotocograph or to reproduce in the most accurate way the related signal. In some approaches, a mathematical model for the simulation of early decelerations in the CTG during birth has been developed [26,27,28]. These studies gave rise to the development of specific software aimed at accurately reproducing the fetal phonocardiographic recordings for testing the FHR extraction algorithms. Other studies have explored the possibility of testing and simulating devices for Doppler-based FHR monitoring [29]. Since the Doppler ultrasound fetal heart signals are weak and complex, while the background noise is strong, some devices can have problems for FHR monitoring. This problem can be addressed by using specific processing algorithms, which include adaptive denoising methods based on spectral subtraction and nonlinear self-excitation suppression processing algorithms. All the proposed algorithms cannot be easily deployed on a hardware simulator since they exploit on the use of previously collected data or are not based on a real time approach. The simulation of fetal phonocardiograph recordings for testing of FHR extraction algorithms [26] can be achieved:

- by modeling non-invasively recorded maternal and fetal Doppler cardiotocography from electrocardiographic signals [6]; and
- by tapping the probe by finger to test the functionality of the probe only (service and tests).

Unfortunately, none of the current solutions is suitable for testing systems as a whole from the probe side to the output of an audible fetal frequency presentation. 

The aim of our work is to design and test a device for simulating the fetus and the mother heart mechanical activity [30,31]. Simulation is important for testing the credibility, accuracy of commercial equipment, and understanding products functionality.

In this context, we propose a novel approach for the design and implementation of an automatic hardware device for testing commercial ultrasound probes commonly adopted for fetal heart rate monitoring. The presented device is low cost and easy to implement since it relies on the use of existing solutions and it does not require the ad-hoc development of new hardware components. The importance of such a system is that it will greatly ease the testing phase of ultrasound probes since it does not require the presence of patients. Moreover, it is fully compliant with the accuracy required by existing technical standards for fetal heart rate monitors [32]. 

The proposed device is similar in concept to the “finger tapping” used to test if an ultrasound probe responds to a mechanical/acoustic input [33]. Our device allows the automatization of this operation with a fixed and reliable reference in terms of multiple frequencies. Since “finger tapping” is very often used to check the probes functionality, we propose a simple and reliable device that can be used as a convenient reference for testing FHR monitor devices. Moreover, the newly designed and realized device allows testing the whole FHR measurement chain, from the points of view of both the HW and SW.

## 2. Materials and Methods

All the instruments used to achieve an FHR signal have to be considered medical devices; consequently, they have to be managed under specific rules also concerning sales and service. To meet all requirements of MDD93/42/EEC and MDR 2017/745, a CTG device must be validated in terms of overall functionality and safety; thus, after the manufacturing, a final inspection test is mandatory, and, during its lifetime, regular safety checks are needed. The specific tests necessary to verify the device safety and its correct functionality are described in IEC 60601-2-27: 201.

### 2.1. The Design of the Fetal Heart Rate Simulator

**Figure 2:**
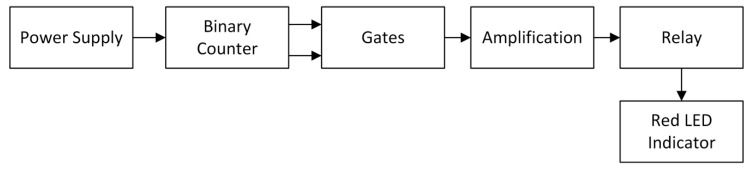
Block diagram of FHRS.

Due to the requirements reported in the mentioned standard, an FHR Simulator (FHRS) was designed and developed to be able to test the correct and accurate functionality of an FHR monitor. The device consists of different elements, such as a power supply, a binary counter, a block of gates, an amplification stage, and an actuator, i.e., sided by a light panel indicator to monitor its specific state. The connections among all the elements are schematized in the block diagram reported in Figure 2, which gives a general idea of the device functionality.

**Figure 3:**
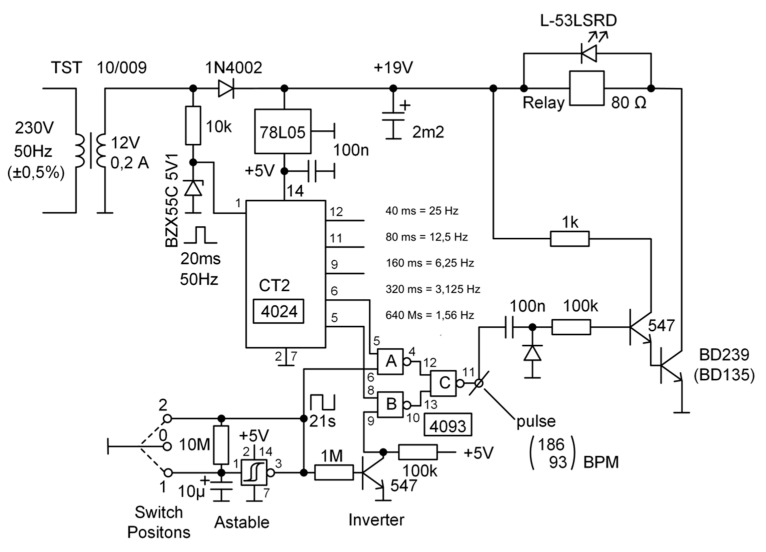
The general scheme of the electrical circuit for the realized FHRS.

The electrical circuit of the developed device is reported in Figure 3: a binary counter (i.e., the CT2 4024, Texas Instruments Inc., USA) is used as a frequency divider to obtain submultiples of two from a main driving clock of a 20-ms period (i.e., obtained from the 50-Hz AC power supply). In this way, it is possible to obtain, among all the possibilities, the two stable clocks whose frequencies are used to drive the actuator in simulating the heartbeats occurrences. The chosen options for the clock multiply are the 2^4^ and 2^5^ ones: in this way, using a driving clock of 20 ms, it is possible to obtain two waveforms with a nominal periods of 320 and 640 ms that correspond to the frequencies of the nominal values of 3.125 and 1.56 Hz. The accuracy in generating the periodic waveforms depends on the accuracy in counting the driving waveform pulse, which, for the CT2 4024, is of about 12 ns: this produces a maximum nominal error of about 0.4 µs for the worst case (i.e., the 2^5^ multiply), thus not altering in a significant way the nominal frequency values. Moreover, the generated frequency accuracy depends on the driving waveform accuracy, that is related to the power supply. In Europe, the accuracy of the AC supply is guaranteed by commercial providers to have a maximum difference of 0.1 Hz on the 50 Hz. This produce a theoretical possible variation on the generating frequencies that becomes 1.56 ± 0.005 and 3.125 ± 0.01 Hz. These output frequencies correspond to a simulation of two possible HR used to test external devices: 186 BMP, corresponding to the simulation of a fetus HR, and 93 BPM, corresponding to the mother’s one. The remaining outputs of the frequency divider are not connected: those could be used as alternative sources for signal with higher frequencies (e.g., to be use as alternative combination), but the available values do not represent a possible physiological frequency. A HCF4093 (Texas Instruments Inc., Dallas, TX USA) integrated circuit, consisting of four two-input NAND gate (i.e., four Schmitt trigger circuits), is used to combine the frequency divider output and to produce a signal that flips between the two frequencies: in this way, the simulation of the FHR frequency and the disturbing phenomena, which CTG must distinguish, is obtained. The resulting signal is amplified by a transistor without modifying its frequencies. Consequently, the relay, driven by this signal, will switch according to its frequencies. A red LED has been added to the circuit to have a visible indicator light of the relay activations. The LED is placed parallel to the relay winding indicates tripping and protects the DB 239 (BD135) collector against overvoltage. To test an FHR monitoring device, an ultrasound gel is placed on the relay and the probe of the device is positioned on it. In this way, the switching of the relay can be detected by the generated Doppler effect.

A 3-A transformer 230-V AC to 12 V is used as power supply for the circuit. Then, a voltage stabilizer 78 L05, which produces a stable +5-V DC voltage from 12 V, is used to power the binary counter and the NAND gates IC (i.e., the components 4024 and 4093). Moreover, the 50 Hz signal is used to provide a frequency fixed signal to the binary counter, using a 10-kΩ resistor to reduce the amplitude. As reported above, two pins of the binary counter are used to simulate the necessary frequencies (i.e., pins 5 and 6) and these are connected to the IC 4093 that will flip these two frequencies between pins 12 and 13 (i.e., outputs of 2 NAND gates of four available), driven by the 21-s period generated signal. The IC 4093 can compose the signals at the two frequencies to produce a single combined signal. Moreover, using a circuit scheme using the connections reported at pins 6 and 9, the signal at pin 11 will result amplified and a composition of the two frequencies provided to the NAND gates. A second power transistor is then used to activate the relay, which presents a low resistance. For the signal amplification, the two further transistors (i.e., the 547 ones) are used while a third one (i.e., the BD239) is adopted for switching the relay. This is used as a mechanical actuator, so only its power circuit is connected while the other pins (i.e., driven switches) remain disconnected. 

Components that were used in the design are wild spare electronic components. The rectifying diode type 1N4002 realizes the 19 VC DC from 13.5-V AC (secondary transformer coil). The Zener diode BZX55C 5V1 0.5W generates from 13.5-V AC impulses 5 V/−0.5 V for all the 4024 counter excitations. As reported in the circuital scheme, to adjust the voltage for the different components and to proper condition the signal in the circuit, different resistors and capacitors are used. A three-way switch is employed as user interface. It can be positioned in the following way: (a) Position “1”, i.e., a connection of the IC 4024 pin 6 to GND; (b) Position “2”, i.e., a connection of the IC 4024 pin 5 to GND; and (c) Position “0”, no connections of the IC 4024 pins 5 and 6 to GND. These options allow different possibility when using this device to evaluate the measurement efficiency of an FHR monitor system. The astable flip-flop input part is realized with the combination 100-nF capacitor and 100-kΩ resistance that produces impulses 60-ms width simulated chamber contraction time. This is time interval was confirmed by measures achieved during the test on the FHRS, as reported in the results Section 3 (Figure 10). The switch in fact is used as an interconnection for the 3.125- and 1.56-Hz signals generated by the binary counter: the two signals, available, respectively, at its pins 6 and 5, are connected directly to the IC 4093, with the switch on the neutral position “0”, where neither is connected to the ground, thus obtaining the combined flipping signals. If the switch is moved to Position “1”, and pin 6 is short-circuited to the ground, only the pin 5 signal is available at the IC 4093. On the other side, if the switch is moved to Position “2”, only the pin 6 signal is available at the IC 4093. As a result, when testing CTG, different signals can be used to test the device, such as a single frequency signals or a combination of both given by the gates, which as described are connected such that the two signals alternate. Thus, CTG and artifacts, which normally occur during the pregnancy observation, are simulated. Indeed, the CTG pregnant woman includes both the heart signal related to the fetus and the one related to the mother.

### 2.2. Tests

The developed device was tested to verify its functionality and accuracy in simulating the necessary signals, and the realized testing procedure consists on several steps. Initially, a measure of the output frequencies of the FHRS was performed using an oscilloscope. Then, to verify that the simulator produces the desired signals, a test was conducted using three different commercial modes of a common CTG clinical system. Indeed, a real measurement test with a validated medical device is necessary to verify the FHRS functionality and the usability.

#### 2.2.1. FHRS Frequencies Measurement on Oscilloscope

**Figure 4:**
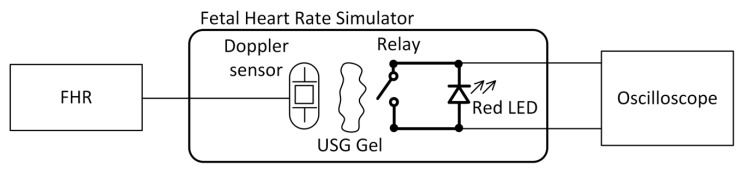
The scheme of the oscilloscope test.

An Agilent MSO-X 2024A oscilloscope equipped with a N2862 probe was used to test the signals generated by the designed device. The adopted connection scheme is reported in Figure 4: since the simulator is designed to produce an acoustic signal related to the effect of the use of an electro-mechanical actuator (i.e., the relay), the oscilloscope was connected directly to the terminals of the red LED. This is connected in parallel to the relay and reproduces its activations during time. Tests were performed setting all the switch positions, and data were directly acquired by the oscilloscope and processed afterwards using MATLAB (R2019b edition, @ MathWorks, Natick, MA, USA).

#### 2.2.2. Measurement on Cardiotocograph with FHRS

**Figure 5:**
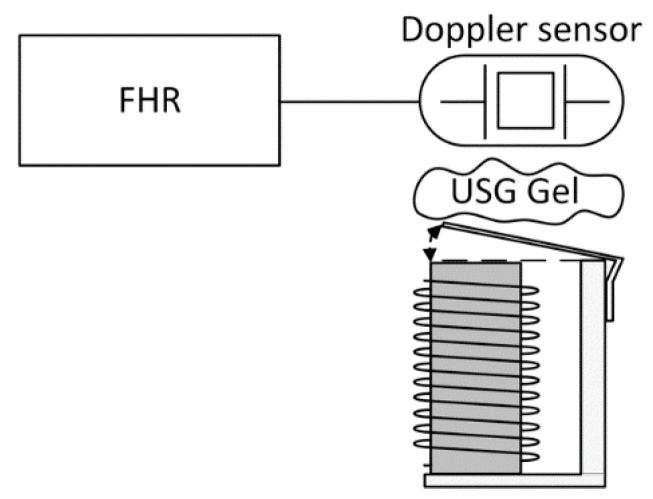
Positioning of the ultrasound CTG probe on FHRS actuator-relay.

Three different CTG monitors that have been certified to be pre-calibrated by the manufacturer were used to achieve measurements from the FHRS, in order to evaluate its stability, once simulation parameters are set, in simulating the heart activities (i.e., fetus and mother signals superimposed). Measurements were performed according the scheme reported in Figure 5: each CTG monitor was coupled with the FHRS using the Doppler probe, coupling this to the relay by using a specific ultrasound gel. 

The tests were performed using three different cardiotocography devices: the CADENCE II (Edan Instruments Inc., Shenzhen, China), the BFM 800 (Bionics Corp Ltd., Honcheon, Korea), and the OCT Baby iFM (ShenZhen Luckcome Technology Inc., ShenZhen, China). The devices were chosen considering different possible application contexts (e.g., intrapartum or outpatient visit) and with different performances, to exploit the FHRM features.

The CADENCE II is a fetal monitor designed for real-time acquisition of the signal and with the possibility of wireless transmission to a remote station. It works with an ultrasound pulse doppler with autocorrelation algorithm and it is able to recognize an FHR in the range 50–240 BPM, with a resolution of 1 BPM and an accuracy of ±1 BPM. It is also equipped with an automatic fetal movement detection procedure and it can be used for intrapartum monitoring.

The BFM 800 is an innovative fetal monitor that allows a continuous real-time recording of the heart rhythm of one or both fetuses (twin births), uterine contractions, event marker, and as an option can provide a fetal ECG. The BFM 800 is normally used for rapid fetal examinations, non-stress tests, and non-invasive monitoring. The capability of fetal heart rate measurement is in the range 50–240 ± 1 BPM. It is standard equipped with two ultrasound probes, one for uterine contractions and one event marker, and powered by a rechargeable battery.

The OCT Baby iFM is a fully portable heart rate monitoring device based on the ultrasound Doppler technology. It is equipped with a Bluetooth connection to communicate with an external device that, using a specific app, can be used as a monitor and to control all the features during the signal acquisition. The capability of fetal heart rate measurement is in the range 50–210 ± 2 BPM, and its reduced weight (85-g battery included) make this device one of the easiest to be used in a wide range of context.

The duration of the test was of 60 s to obtain a significative number of heart activity events: these signals were automatically acquired by each CTG monitor that gives as output the measured heart rate. The measured values were compared with the imposed one, given by the FHRS. Tests were conducted using the same setup designed or oscilloscope measurements, that is changing all the possible switch positions. Each CTG device was used to read the simulated signal for a minimum of three trials to assess the possible difference in BPM readings.

## 3. Results

**Figure 6:**
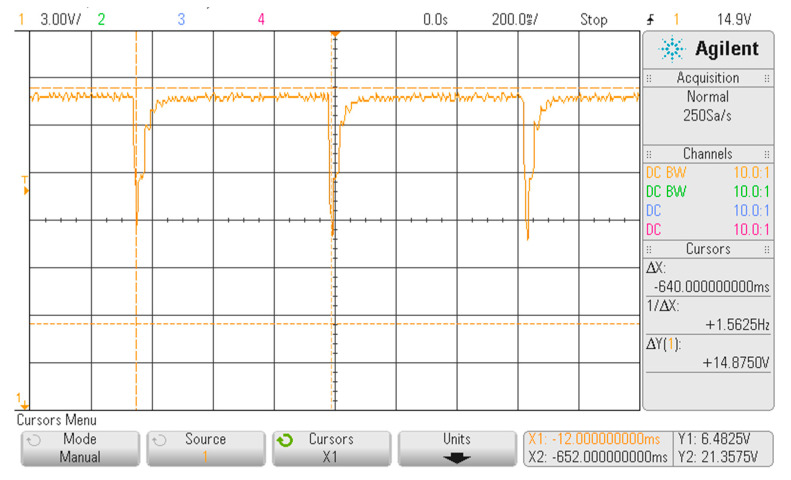
Displayed waveform on the oscilloscope with the FHRS switch in Position “1”.

**Figure 7:**
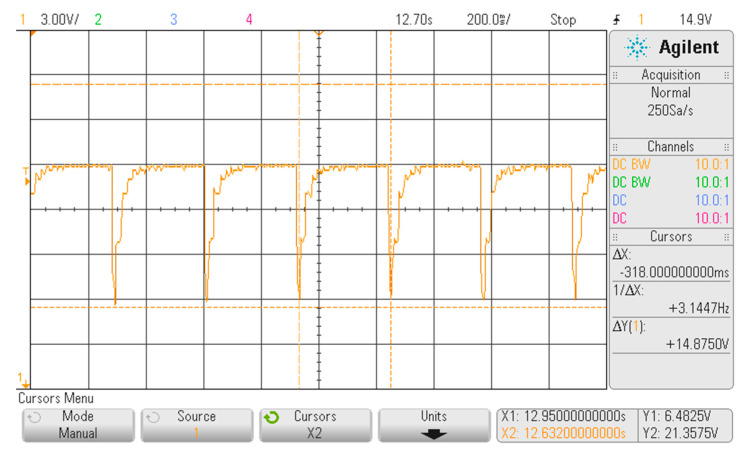
Displayed waveform on the oscilloscope with the FHRS switch in Position “2”.

**Figure 8:**
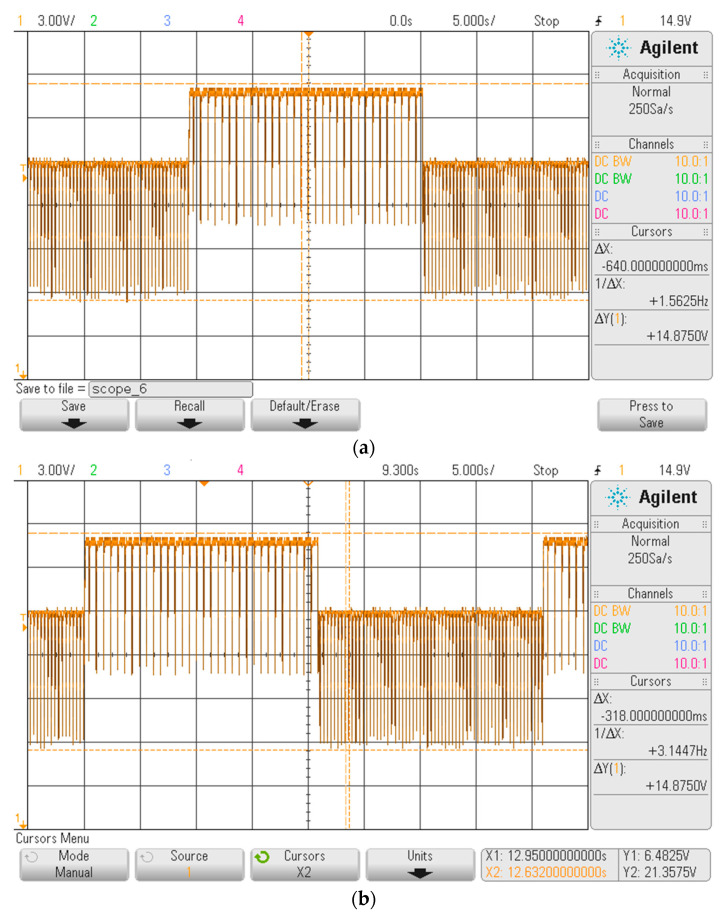
Screenshot from oscilloscope at the FHRS switch position 0. There are visible switched frequencies 1.56/3.144 of simulated signal every 21 s. The cursors were placed on signal period x and recalculated to frequency as 1/∆x: (**a**) measured f = 1.56 Hz; and (**b**) measured f = 3.144).

In Figure 6, Figure 7 and Figure 8, three portions of the signal acquired from the FHRS, useful to evaluate the simulated signal at prefixed frequencies, for all the switch position are reported. As shown in Figure 6, the FHRS was tested moving the control switch to Position “1”, that is when the frequency f = 1.56 Hz is generated. The simulator provides a series of pulsed waves every ∆t = 640 ms: indeed, a mean frequency value of f_m_ = 1.5625 Hz is visible in the display. The pulsed waves are due to the relay activation, derived by the power supply frequency that is 50 Hz: driven by this activation, i.e., modified by the binary counter, the relay produces an acoustic waveform that is used to activate the CTG probe. The measured value is very stable along time since the measured frequency variation is about 1 mHz. Due to noise in the measurement chain, a variation on the amplitude of ∆V = 0.5 V on the “not active” phase of the relay was observed, while a ∆V = 0.2 V on a range of about 10 V in the generated activation waveform during the “active” phase of the relay was observed. Both variations in generating the relay activation waveform do not significatively affect the performance of the FHRS in simulating the desired frequencies since a consistent voltage variation occurs and this drives properly the relay that produces the acoustic signal for testing the CTG In fact, these medical devices should read this specific value with no alteration: if drift in the observed frequency or an unstable value is read by the CTG monitor under test, the analyzed medical device should be considered not properly working and inspected by the maintenance service. 

As shown in Figure 7, the FHRS was tested moving the control switch to Position “2”, that is when the frequency f = 3.125 Hz is generated. The simulator provides a series of pulsed waves every ∆t = 318 ms: indeed, a main frequency value of f_m_ = 3.1447 Hz is visible in the display. In addition, in this case, the pulsed waves are due to the relay activation, obtained by the conditioned power supply frequency that is 50 Hz. About the measured amplitude, still some noise due to the measurement chain was present: an output variation of ∆V = 0.3 V on the “not active” phase of the relay was observed, while a ∆V = 0.3 V on a range of about 10 V in the generated activation waveform during the “active” phase of the relay was observed. Concerning the generated frequency, the visible display value of f_m_ = 3.1447 Hz is a little bit different from the theoretical one provided by the binary counter (i.e., at pin 6 of the IC 4024), which is supposed to be f = 3.125 Hz, with an observed difference of ∆f = 0.0197 Hz. This variation of 0.6% with respect the nominal one is a bias that can be considered a sum of the effect of the uncertainty due to the accuracy of the voltage divider and the stability of the 50 Hz driving waveform. However also in this case, the measured frequency variation during time was less than 1 mHz.

As shown in Figure 8, the FHRS was tested moving the control switch to Position “0”, that is when the generated output signal flips between the two frequencies at f = 1.56 Hz and f = 3.125 Hz. The simulator provides a cycling signal composed by a series of pulsed waves 21 s in duration, followed by another series of pulsed waves every ∆t = 646 ms of 21 s in duration as well. The oscilloscope shows a main frequency of f_m_ = 3.1447 Hz for the first 21 s, followed by a main frequency of f_m_ = 1.5625 Hz for the next 21 s. As regards the measured amplitude, some noise due to the measurement chain was present: an output variation of about ∆V = 0.6 V on the “not active” phase of the relay was observed for both part of the compound signal, while a ∆V = 0.35 V was observed over an interval of about 10 V in the activation waveform generated during the “active” phase of the relay. As far as the generated frequencies are concerned, stable values of the frequencies can be observed in both parts of the generated signal, with accuracy similar to the one obtained in the previous test (i.e., switch in Positions “1” and “2”). The compound signal reproduces very well the condition of a manual CTG exam, where the probe is moved closer to the source of the low frequency signal (i.e., capturing the mother heart rate) or closer to the source of the high frequency signal (i.e., capturing the fetus heart rate).

A summary of obtained values is reported in Table 1: for all switch positions, the theoretical simulated values are reported, together with the measured values and the correspondent heart rate. 

**Figure 9:**
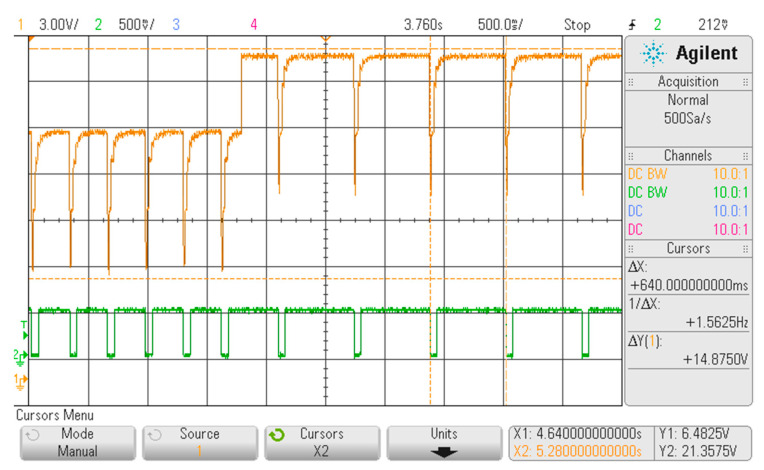
Displayed waveform on the oscilloscope with the FHRS switch in Position “0”. The yellow curve represents the signal measured on primary coil of relay at set on frequencies. The green curve shows signal measured on switched electrical part of relay, which is realized by mechanical contact of relay tab.

**Figure 10:**
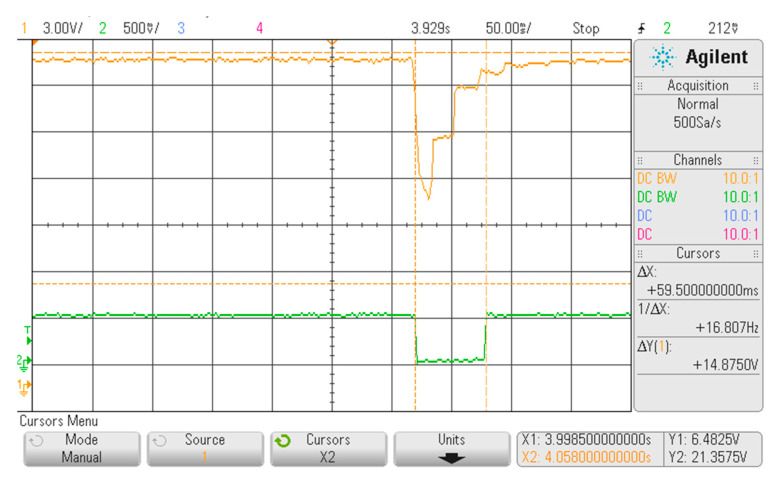
Detail of Figure 10, representing the transition phase when the relay is excited.

The correspondence in relay activation and pulsed waves was assessed by measuring the signal at its primary coil and the signal that is obtained in the output switch when connected to a measuring circuit: in Figure 9, this correspondence is well remarked, where the device was tested with switch in Position “0”. The output signal (green) is not depending on the exciting input amplitude, but only by its frequency, thus guaranteeing accurate FHR values during the simulation. As reported also in Figure 10, the activation of the relay is constant during all the activation cycle, representing one heartbeat: a duration of 60 ms of the wave, corresponding to the same time before releasing the relay tab during mechanical switch, is obtained both in generating the set waves at 1.56 and 3.12 Hz. This time well represents the physiological phenomena, since it corresponds to the heart muscles contraction duration [34].

To verify the FHRS functioning in an “on the field” condition, a measurement test was performed using the three different CTG devices described above: CADENCE II, BFM 800, and OCT Baby iFM. Even if the three CTG devices are based on the same technology, they are designed for different scenarios, such as installed in the surgery area or used for clinical diagnosis. In addition, the performance of the considered devices is different (e.g., accuracy, range, etc.) and related to this aspect the purchasing cost is different.

The FHRS was tested on all devices and no problems were highlighted for the physical compatibility with their probes: all adopted test CTG devices can be used on the FHRS using the scheme reported in Figure 5, since the size of the mechanical actuator (i.e., the relay) and the probes is compliant. As for the BPM detected by each of the three CTG devices, the results obtained, as shown in Table 2, in all three positions of the FHRS switch (i.e., Positions “1”, “2”, and “0”) show that a maximum difference of 2 BPM is found in Positions “1” and “2” of the switch, while a maximum difference of 1 BPM is found with the switch set to Position “0”. The repeated measure in the same switch position shows a constant value of the readings, with no variations of the displayed BPM on the CTG device.

## 4. Discussion and Conclusions

This work deals with the design and realization of novel equipment for testing fetal heart rate simulator device, verifying their capability in fetus heart rate detection. Since the fetus heart rate is in the range from 90 to 190 BPM, the FHRS can also be used for testing the limit values of these conditions. The chosen frequencies were set to simulate the simultaneous presence of the mother and fetus heart activities, thus the FHRS was designed to allow flip between these frequencies. To obtain these values, as described above, two outputs of the binary counter (i.e., outputs 5 and 6) were used: these outputs correspond to the testing frequencies of 1.56 and 3.125 Hz, indicating a heart rate activity of 93.6 and 187.5 BPM, respectively. The obtained signal results very stable and not affected by external interferences or technical artifacts (i.e., the 50 Hz signal of the electric net). 

Using a different solution in frequency defining, more and different values can be simulated. However, it is important to highlight that with the adopted solution (i.e., use of the selected binary counter) it is possible to test the capability of an FHR device to distinguish two close heartrate values. This is important because, since these values represent typical ones for fetal heart rate monitor device exams, the medical device could have some limits in recognizing such kind of close frequency values, because of possible limits in recognition algorithm. The FHRS is thus a valuable tool to test this specific feature in fetal heart rate monitor device based on ultrasound.

**Figure 11:**
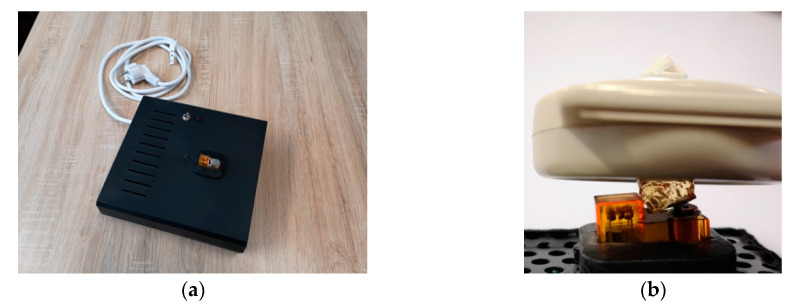
Final prototype of the fetal heart rate simulator device: (**a**) fetal heart rate simulator device. and (**b**) relay and US probe during simulation tests.

Moreover, on the constructive point of view, it is also important to highlight that the proposed device can be used for prolonged test times (i.e., more than 2 h) since it has not shown any overheating during long-term tests. Indeed, if a simulator has heating problems, it could produce not neglectable noise so that the SNR of the output signal could be badly affected. The power supply and electronic board was placed in a box that presents on the bottom side the relay. The LED reported out of the box is a useful tool for a rapid inspection of the FHRS functioning conditions, as visible in Figure 11. 

This is, as described above, a mechanical actuator and is used as heart mechanical simulator: this configuration allows to use the designed FHRS as a “patient simulator” during the fetal heart rate monitor devices tests. In fact, no additional devices or adaptors are needed in testing a device, thus reducing the testing duration and simplifying the setup. Currently, there are guidelines for testing the functionality of CGT that are based on checking that the device is properly working and that it respects the requirements for electric safety, as for a generic medical device. Typically, these tests are performed to highlight the presence of faults before the device is commercialized. However, there are no guidelines for testing the correct functionality of the device during its lifecycle. 

The presented device is reliable and easy to implement since it is based on the use of existing solutions and it does not require the ad-hoc development of new hardware components, only a circuit customization. To the best of our knowledge, no similar solutions are available in the commercial or research field. The importance of such a system is that it will greatly ease the testing phase of ultrasound probes since it does not require the presence of patients. Moreover, it is fully compliant with the accuracy required by existing technical standards for fetal heart rate monitors. All these aspects could contribute to the reduction of maintenance frequency, since this device allows implementing in a simple, quick, and reliable way a FHR monitor and CTG devices test directly where it is located, with positive consequences in terms of costs reductions and increased time of availability of the medical devices. The use of simulator in such kind of contexts has largely been adopted for other systems such as ECG simulators for ECG devices.

The tests performed on the adopted FHR devices showed a practical convenience in the use of the FHRS to simulate a stable signal. The minimum variations are due to the conditions of the test executions: indeed, the test performed with oscilloscope were conducted connecting a fixed probe to the electrical pins of the relay and showed a stable signal over time, while, when using a FHR, a “manual” operation is executed in applying the ultrasound probe to the surface of the relay actuator, thus giving rise to the possibility of having small variations on the readings obtained on the FHR. Moreover, these small variations are due to the rounding of the calculated BPM by the FHR devices: the BPM computational algorithm gives an integer value on the display, since a decimal value does not have a significance while it is used in clinical diagnostics by the final users. 

The use of the FHRS will allow a valid and reliable tool that could also be used for re-calibrating all the system used to monitor the FHR, thus increasing the accuracy and precision of such kind of device in the heart activity monitoring. 

Further possibility of simulating signal can be added to the developed system, obtaining for example more FHR frequencies in the generated signal, or including all the possible disturbance signals, that can help evaluating the performances of FHR and CTG devices in revealing the correct FHR. This could be obtained with a digital controller that could drive the generation of a signal at specific frequencies, which could be selected using dedicated software. In this application, some extras could also be added, such as the possibility of changing the SNR of the signal or adding some artifact, thus creating a wide scenario of possible tests for FHR and CTG devices.

All implementations achieved in this project and all the design features are fully compliant with the IEC standard for measuring CTG instruments, thus the FHRS is potentially ready to be used in clinical environments or by the companies producing or servicing medical devices. 

## Figures and Tables

**Table 1:** Measured parameters of FHRS on oscilloscope.

Switch Position	1	2	0
binary counter settings (input @50 Hz)	640 ms (1.56 Hz)	320 ms (3.125 Hz)	320 ms and 640 ms
Measured frequency (Hz)	1.562 ± 0.001	3.144 ± 0.001	1.562 ± 0.001 and 3.144 ± 0.001
correspondent BPM (beat/min.)	93.72	188.6	93.72 and 188.6

**Table 2:** The comparison outputs of the designed mechanical heart rate mechanical simulator device and settings values.

	Switch Position (Simulated BPM)		
Ctg Device and Corresponding Average Measured Bpm	1 (93 BPM)	2 (186 BPM)	0 (test on the low frequency part @93 BPM)
CADENCE II	95	188	94
BFM 800	93	187	93
OCT Baby iFM	94	186	94
